# 1831. Beyond the Viral Load: Infectious Disease Clinicians as Primary Providers for People Living with HIV

**DOI:** 10.1093/ofid/ofad500.1660

**Published:** 2023-11-27

**Authors:** Isaac Daudelin, Tanzila Salim, Sahiba Khurana, Ashish Murthy, Steven Keller, Diana Finkel, Eli S Goshorn

**Affiliations:** Rutgers NJMS, Union, New Jersey; NJMS RUTGERS, Newark, New Jersey; Rutgers New Jersey School of Medicine, Jersey City, New Jersey; Rutgers New Jersey Medical School, Hillsborough, New Jersey; Rutgers New Jersey Medical School, Hillsborough, New Jersey; Rutgers NJMS, Union, New Jersey; Rutgers New Jersey Medical School, Hillsborough, New Jersey

## Abstract

**Background:**

With advances in antiretroviral therapy, life-expectancy for people living with HIV (PLWH) has increased. Many HIV specialists function as primary care providers for PLWH and are often the front-line of defense when it comes to preventative healthcare. The Infectious Disease Practice (IDP) at University Hospital in Newark, NJ serves as a medical home and primary care site for many PLWH. The clinic incorporates preventative healthcare measures into providing whole patient care as well as focusing on HIV specific care. Here, we present data from multiple quality assessment projects at our clinic assessing preventative health measures in PLWH.

**Methods:**

Following IRB NHS determination, data was collected from an EPIC database of PLWH seen at IDP. Guidelines for Primary Care by Infectious Disease Society of America, the Advisory Committee on Immunization Practices, JNC8, and the US Department of Health and Human Services were reviewed.

**Results:**

Bone mineral density (BMD) screening was ordered for 59% and completed in 39% of PLWH who met guideline criteria compared to national average of 7.4%

Among PLWH with Diabetes mellitus (DM), 62% achieved A1C goals, 65% achieved blood pressure goals and 67% achieved LDL-c goals. National averages for achieving A1C, blood pressure, and LDL-C goals in people with DM were 51%, 70%, and 66% respectively in 2018.

Hypertension pharmacologic therapy was initiated, and blood pressure controlled to systolic blood pressure < 150 diastolic blood pressure < 90 in 72% of PLWH compared to national average of 65%

Among PLWH, 64% received at least one dose of PCV13, 60% received at least one dose of PPSV23, and 38% were fully up to date compared with national average 23% in PLWH in 2018 receiving at least one dose of either vaccine.

**Figure:**
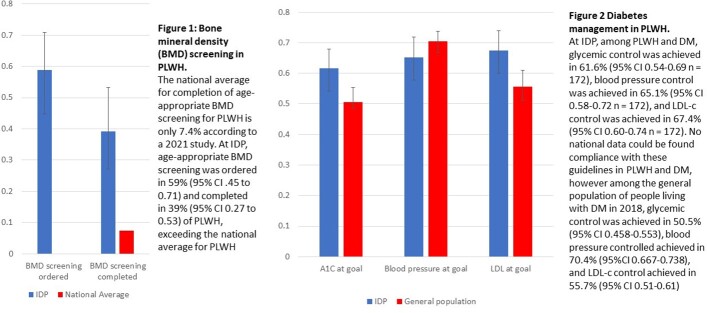

**Figure:**
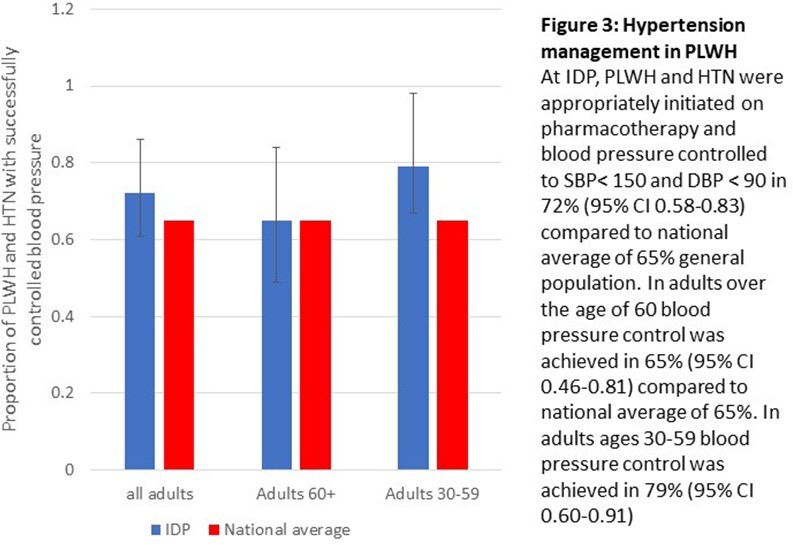

**Figure:**
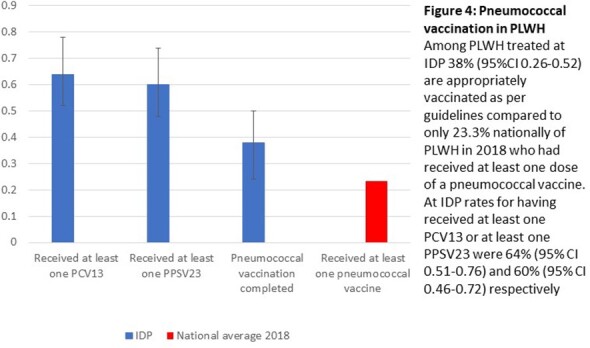

**Conclusion:**

Clinicians practicing in HIV clinics should be up to date on age-appropriate screenings and vaccinations. The IDP has matched or outperformed national standards in several aspects of preventative healthcare for PLWH including DM, hypertension, vaccinations, and BMD screening. Infectious disease specialists who provide care for PLWH are in a unique, trusted position to deliver preventative healthcare and should have expertise in primary care to optimize patient centered care.

**Disclosures:**

**Eli S. Goshorn, MD**, Genmark Diagnostics Inc: Advisor/Consultant

